# Demographic, Behavioural and Anthropometric Correlates of Food Liking: A Cross-sectional Analysis of Young Adults

**DOI:** 10.3390/nu12103078

**Published:** 2020-10-09

**Authors:** K.M. Livingstone, H. Pnosamy, L.J. Riddell, S. Cicerale

**Affiliations:** 1 Institute for Physical Activity and Nutrition (IPAN), School of Exercise and Nutrition Sciences, Deakin University, Geelong 3220, VIC, Australia; lynn.riddell@deakin.edu.au; 2 CASS Food Research Centre, School of Exercise and Nutrition Sciences Deakin University, Deakin University, Geelong 3220, VIC, Australia; hpnosamy22@gmail.com (H.P.); js.fieschi@gmail.com (S.C.)

**Keywords:** food liking, diet quality, dietary guideline index, body mass index, health behaviours, correlates, young adults, Australian

## Abstract

The degree to which foods are liked or disliked is associated with dietary intake and health behaviours. However, most food liking research has focused on single foods and nutrients and few studies have examined associations with demographics and health behaviours. Thus, this study aimed to investigate the association between food liking and socio-demographics, health behaviours, diet quality and body mass index (BMI) in a sample of young Australian adults. Data from 1728 undergraduate students (21.8 (standard deviation [SD] 6.0) years; 76% female) were used. Food liking scores and a diet quality index (Dietary Guideline Index, DGI) were estimated from a Food Liking Questionnaire and Food Frequency Questionnaire (FFQ), respectively. Multivariate linear regression analyses were used to assess the association between food liking and correlates. Young adults with higher liking for encouraged core foods were older, female, did their own food shopping, consumed less packaged foods and had better diet quality. Higher liking for discretionary foods and beverages was associated with less healthy behaviours, such as smoking, higher BMI and lower diet quality. These results suggest that food liking measures may offer an appropriate methodology for understanding influences on young adults’ food choices, adding to the body of literature investigating the potential for food liking scores to assess diet–disease relationships.

## 1. Introduction

As over 50% of young Australian adults aged 18–30 years are overweight or obese [1], poor diet is considered the leading risk factor for disease in this age group [2,3]. To improve the diet and health of young adults, it is first necessary to understand the influences on their food choice [4]. Within the framework described by Furst et al. [5], life course influences, such as personal and social influences, and value negotiations, such as sensory perceptions and health and nutrition, shape food choice and intake. Moreover, how much a food is liked or disliked, i.e. food liking, is considered a central construct in many hypothetical models of food choice [5,6,7]. However, most food liking research has focused on associations with dietary intake and health outcomes [8,9,10,11] and has been conducted in specific institutions, such as the army [12,13] or hospitals [14], and few studies have examined how food liking is correlated with other influences on food choices, such as demographics and food behaviours. Understanding these correlates in young adults is necessary to inform the effective design of research using food liking to assess diet–disease relationships.

Young adulthood is a life stage characterized by life transitions, including becoming responsible for food purchasing and preparation [4]. The transition to university has been shown to negatively impact on food choices, particularly among those who live away from home [15,16,17,18]; thus, these changes may disproportionately influence the relationship between food liking and food intake. While young adults report higher liking of discretionary foods, i.e., foods high in added-salt, sugars and fat, than encouraged core foods, i.e., fruits and vegetables [9], other personal, social and environmental factors may influence the relationship between food liking and subsequent food intake. The development of Food Liking Questionnaires has provided an opportunity to explore the correlates of food liking in larger samples than is possible using laboratory based sensory testing [8,12,19,20]. However, there is a paucity of studies that have investigated a wide range of food liking correlates in young adults at university [9], and none have investigated food literacy behaviours, such as cooking and food shopping. Moreover, most studies have focused on liking of nutrients (i.e. fat, salt and sweet) rather than food groups [21,22].

Given that foods and nutrients are not eaten in isolation, an increasing body of research is focusing on overall diet quality indices, which score intakes of multiple foods and/or nutrients against recommended intakes [23,24,25,26]. To date, only one study has examined the associations between liking specific food groups (e.g. fruits, vegetables and discretionary foods) and overall diet quality [9]. Prior to implementing and translating food liking scores for assessing diet–disease relationships in young adults, a broader understanding of the correlates of liking for specific food groups is needed [4]. Moreover, the relationship between liking specific food groups and a broad range of university-based young adult demographics and health behaviours, such as living situation, indicators of socio-economic status and food literacy behaviours, is unclear. With the high prevalence of obesity, being overweight and unhealthy food choice behaviours in young adults, there is also a need to investigate the dietary and anthropometric correlates of food liking in this at risk age group. It is hypothesized that young adults with higher liking for discouraged foods (e.g., foods high in added salt and sugars) will be younger, male, have less healthy behaviours, lower diet quality and higher body mass index (BMI). Thus, the aim of this study was to investigate the association between liking specific food groups and demographics, health behaviours, diet quality and BMI in a young adult cohort.

## 2. Materials and Methods

### 2.1. Study Design and Participants

Cross-sectional data were collected from undergraduate students enrolled in a food and nutrition unit at Deakin University (Victoria, Australia) within the period 2015–2017. Students were invited to participate in class and were asked to complete three online surveys as part of their assignment: a FFQ, Food Liking Questionnaire, and Food and Diet Questionnaire [9,19]. A total of 2070 participants provided written consent (82% response rate) for their data to be used for research purposes. Participants were excluded if they did not complete all three questionnaires and if they provided missing information on their weight and height. All participants gave their informed consent for their data to be used for research purposes. The study was conducted in accordance with the Declaration of Helsinki, and the protocol was approved by the Human Research Ethics Committee of Deakin University (EC 163-2009).

### 2.2. Questionnaires

The semi-quantitative FFQ used in the study was adapted from the 1995 National Nutrition Survey [27]. The questionnaire was based on an existing validated FFQ developed for the Australian population that has been used in Australian young adult cohorts [9,16]. The FFQ assessed the frequency of consuming selected foods, beverages, and vitamin and mineral supplements over the prior month, with options ranging from “never or less than once a month” to “six or more times per day”. A total of 133 food items were included from across 10 categories: dairy foods, bread and cereal foods, meat, fish and eggs, sweets, baked goods and snacks, dressings, non-dairy beverages, vegetables consumed as a side dish/in mixed dishes, vegetables, fruits, and dietary supplements.

The Food Liking Questionnaire was based on a validated Food Liking Questionnaire for use in adult cohorts of similar age and demographic profile [8]. Moreover, it has been previously used and validated in young Australian adults [9,19], with test–retest reliability of moderate to excellent [19]. The questionnaire was adapted for foods available in Australia, which included the addition of culturally specific foods such as Tim Tams, Kentucky Fried Chicken, rotisserie chicken and cornflakes. The majority of foods in the FFQ also featured in the Food Liking Questionnaire. The questionnaire contained 73 food items and measured liking using a nine-point hedonic scale representing degrees of liking from ‘dislike extremely’ to ‘like extremely’. Food liking scores were derived from the Food Liking Questionnaire. As shown in Table S1, the foods in the Food Liking Questionnaire were grouped into a total of 12 groups according to the 2013 Australian Dietary Guidelines to create overall food liking scores for each group [28]. For example, a group for ‘grains’ was created that included food items such as porridge, muesli, bread, spaghetti and rice, and a group for ‘dairy’ included food items such as milk, yoghurt and cheese [9,19]. Information on food items within each food group and correlations between food groups are presented in Table S2. Eight single food groups were created: grains, vegetables, fruits, plant-based proteins, animal-based proteins, dairy, alcohol, and fats and oil. An ‘encouraged’ core food group representing foods encouraged in the Australian Dietary Guidelines was produced by aggregating grains, vegetables, fruits, plant-based proteins, animal-based proteins, and dairy. A total discretionary food group was created, as well as two sub-groups (salty discretionary foods and sweet discretionary foods). The internal reliability of these 12 food groupings was estimated using Cronbach’s alpha. The Cronbach’s alpha values were interpreted as unacceptable (<0.50), poor (0.51-0.60), questionable (0.61-0.70), acceptable (0.71-0.80), good (0.81-0.90), and excellent (0.91-1.00) [29]. Information on these grouping and the internal reliabilities are presented in Table S1.

The Food and Diet Questionnaire has been used in previous studies [15,16] and is based on questionnaires previously used to investigate the eating habits of young adults [30,31]. The Food and Diet Questionnaire collected information on age, sex, nationality (free text), highest level of maternal and paternal education (no formal qualifications, Year 10 or equivalent, Year 12 or equivalent, trade/apprenticeship, certificate/diploma, university degree, higher university degree), smoking habits (yes, no), living arrangements (with parents, flatmates, partner, by self), doing own food shopping and cooking (yes, no), frequency main meal is convenience or packaged (e.g. canned stew, frozen dinner/pizza, packet noodles) (6 point scale from never to 6-7 meals/week or more), self-reported health of diet (very unhealthy, unhealthy, healthy, very healthy), and weight and height [16]. BMI (kg/m^2^) was calculated from the height (cm) and weight (kg) of participants and classified into three groups according to the World Health Organization (WHO) criteria: underweight (≤18.5kg/m^2^), normal weight (18.5–24.9kg/m^2^) and overweight/obese (≥25kg/m^2^) [32]. Due to low numbers is some categories, nationality was dichotomised into Australian and non-Australian; education was collapsed into low (no formal qualifications, Year 10 or equivalent, Year 12 or equivalent), middle (trade/apprenticeship, certificate/diploma) and high (university degree, higher university degree); living arrangements were dichotomised into living at home and living away from home; frequency main meal is convenience or packaged was dichotomised into less than one/week and more than once/week and self-reported healthy diet was collapsed into healthy and very healthy.

### 2.3. Diet Quality

The dietary guideline index (DGI) was calculated from the FFQ. The DGI is a food-based score designed to reflect the overall diet quality of individuals according to the 2013 Australian Dietary Guidelines [26,33]. This score has been used previously in Australian adult populations and is described in more detail elsewhere [25,34,35]. Briefly, the DGI score was made up of 13 components: seven encouraged food groups (diet variety, fruits, vegetables, grain foods, lean meat and alternatives, dairy products and water) and six discouraged food groups (discretionary foods high in saturated fat, added salt, added sugars and alcohol, foods high in saturated fat, small allowance of unsaturated fats or spreads, foods and drinks high in added salt or added sugars and alcohol). Each component contributed a maximum of 10 points toward the total DGI score (0 indicating guideline not met, 10 indicating guideline met) and included age and sex-specific cut points in accordance with the Australian Dietary Guidelines. The DGI score ranged from 0-130 points, with a higher DGI score indicating a better diet quality.

### 2.4. Statistical Analysis

A complete case analysis approach was used. Numerical data was expressed as means and standard deviation (SD) while categorical data was expressed as percentage (%). Descriptive statistics were used to describe participant characteristics. Multiple linear regression analyses were used to examine the association between each food liking score (dependent variable) and all correlates (independent variables): age (continuous), sex, nationality (categorical), highest level of maternal and paternal education (categorical), smoking habits (binary), living arrangements (categorical), doing own food shopping and cooking (binary), frequency main meal is convenience or packaged (binary), self-reported health of diet (binary), diet quality (continuous) and BMI (continuous). Multicollinearity was assessed using variance inflation factors (VIF). Linearity was assessed by visual inspection of added variable plots and by adding squared terms for independent variables to the model to allow for quadratic effects. No evidence of multicollinearity (VIF <4.0) and non-linear relationships (*p* < 0.05) were observed (data not shown).

As a sensitivity analysis to help understand the relationship between food liking, dietary intake and BMI, we examined concordance (i.e., reported higher liking and high intake) and discordance (i.e., reported higher liking but lower intake) between food liking from the Food Liking Questionnaire and food intakes from the FFQ for encouraged core foods and discretionary foods. For liking of encouraged core foods, total DGI score was used. For liking of discretionary foods, the DGI sub-group for discretionary foods was used. Two-sample t-tests were used to investigate the mean differences in BMI between concordant and discordant groups for encouraged core foods and discretionary foods. Data analysis was performed using Stata (StataCorp. 2019. Stata Statistical Software: Release 16. StataCorp LLC, College Station, TX).

## 3. Results

Of the 2524 students enrolled in the undergraduate cohort, 2070 participants provided written consent to participate (82% response rate). A total of 342 participants (17%) were excluded for a combination of incomplete FFQ and Food Liking Questionnaires (*n* = 97) and missing data on demographics (*n* = 197), health behaviours (*n* = 128) and height and weight (*n* = 106). A total of 1728 participants were included in the present analysis. Demographic characteristics were comparable between participants who were included compared to those who were excluded (data not shown). As shown in Table 1, the majority were female (76%), of Australian nationality (83%) and living at home (72%). Thirty-eight percent of young adults had highly educated mothers, while 36% had highly educated fathers. Five percent were smokers, with less than half doing their own food shopping (46%), cooking their own meals (21%) and never eating convenience or packaged foods for their main meal (42%). The majority of participants rated their diet as healthy (67%). The mean BMI was 22.9 (SD 3.5) kg/m^2^ and mean DGI was 94.2 (12.3).

### 3.1. Food Liking and Diet Quality

The internal consistency scores of the 12 food liking groups are shown in Table 2. The highest internal consistency was for total discretionary foods (α = 0.92), while the lowest was fats and oil (α = 0.49). Mean diet quality was 94.0 (SD 12.5). As shown in Table 2, food liking scores for encouraged core food groups ranged from 6.18 for plant-based protein to 7.60 for fruit, indicating a slight-to-moderate liking for encouraged core foods. Discretionary foods (including sub-groups for high salt and added sugars) were liked slightly, while alcoholic beverages were disliked slightly and fats and oil were neither liked nor disliked.

### 3.2. Correlates of Food Liking

As shown in Table 3, Table 4 and Table 5, higher age was associated with higher liking for total encouraged core foods, grains, vegetables, legumes and plant-based alternatives, meat and animal-based alternatives, dairy, alcoholic beverages, and fats and oil. Compared to males, females had a greater liking for grains, vegetables, fruit and legumes, and plant-based alternatives, and a lower liking for meat and animal-based proteins, dairy, discretionary foods, alcoholic beverages, and fats and oil. Young adults of Australian nationality had a lower liking for fats and oils compared to non-Australian nationalities. Young adults living away from home had a higher liking for total encouraged core foods, grains, fruit, dairy and alcoholic beverages compared to participants living at home. Young adults with higher maternal education had a lower liking for meat and animal-based alternatives, while those with higher paternal education had a higher liking for alcoholic beverages. Young adults who were smokers had a higher liking for alcoholic beverages. Higher BMI was associated with lower liking for vegetables and legumes and plant-based alternatives, and a higher liking for meat and animal-based alternatives, dairy, discretionary foods and fats and oils. Young adults who did their own food shopping had a higher liking for total encouraged core foods, grains, fruit, meats and animal-based alternatives, dairy, discretionary foods and fats and oil. Young adults who did their own cooking had a lower liking for total encouraged core foods, vegetables, legumes and plant-based alternatives and fats and oil. Young adults who consumed packaged foods more often had a lower liking for total encouraged core foods and vegetables and a lower liking for discretionary foods and fats and oils. Young adults with higher self-reported healthy diets and diet quality had a higher liking for total encouraged core foods, grains, vegetables, fruit, legumes and plant-based alternatives, and a lower liking for discretionary foods and fats and oils (Table 3, Table 4 and Table 5).

### 3.3. Concordance and Discordance

Fifty-nine percent of young adults were concordant for total encouraged core foods, i.e., reported both higher liking (from the Food Liking Questionnaire) and intakes (from the FFQ) of encouraged core foods. There was no evidence of a mean difference in BMI between participants who were concordant and discordant (i.e., reported higher liking but lower intake) for liking of encouraged core foods (*p* = 0.42). In contrast, participants who were concordant in discretionary food liking and intake (i.e., reported higher liking and higher intake) had a higher BMI compared to those who were discordant (22.7 kg/m^2^ vs 23.2 kg/m^2^; *p* = 0.003).

## 4. Discussion

The present study aimed to examine the demographic, behavioral and anthropometric correlates of food liking in a young adult cohort. Our main findings are that young adults with higher liking for encouraged foods were older, female, had healthier behaviors (including food shopping and consuming less packaged foods), and better diet quality. In contrast, higher liking for discouraged foods and beverages was associated with less healthy behaviours, such as smoking, higher BMI and lower diet quality. These results suggest that food liking measures may offer an appropriate methodology for understanding influences on young adults’ food choices, adding to the body of literature investigating the potential for food liking scores to assess diet–disease relationships.

The present study identified demographic correlates of food liking. A greater liking for encouraged foods with higher age and in females is consistent with previous research [31,36,37,38], which could be attributable to females and older adults being more health and weight conscious and having more knowledge about nutrition in general [39]. In contrast to previous research, there was limited evidence that nationality was a correlate of food liking and findings suggested that living away from home was associated with a higher liking for encouraged foods, with the exception of a higher liking for alcoholic beverages [15]. Given that research suggests that young adults adopt unhealthier food choices when living away from home [4,15], our results may be partly attributable to the female-skewed sample and social desirability biases associated with participants being from a nutrition course. Nonetheless, with research suggesting there are different biological and/or behavioral drivers of food choice between males and females [40], these findings add to the body of literature supporting the tailoring of dietary interventions by sex.

Comparable to previous research, the present study identified that food liking was associated with dietary intake [9,10,14,37]. Most research to date has focused on single foods and nutrients, where higher liking for high-fat foods and fatty sensations has been associated with higher intake of total energy and saturated fat, as well as higher intake of meat and discretionary foods. Further, higher liking for high-fat and high-salt foods and their associated sensations has been associated with higher intakes of sodium and alcoholic beverages [8,37]. Given the complex nature of food choice [5], increasing research is focusing on overall diet quality indices, which, consistent with the present study, has shown that higher liking for encouraged foods is associated with better diet quality [9,14]. These findings demonstrate the potential for using simple and low participant burden food liking measures as dietary assessment tools [8].

Few studies have examined the behavioral and anthropometric correlates of food liking in young adults. This information is needed to inform the effective use and implementation of food liking scores to assess diet–disease associations. Food involvement and confidence research suggests that food shopping and cooking behaviors are important predictors of food choice [41]. Our findings add to this body of literature by demonstrating evidence for associations between liking encouraged foods, young adults doing their own food shopping and cooking, and consuming less packaged foods. Evidence for higher liking of alcoholic beverages in smokers, lower liking of encouraged foods, and higher BMI is consistent with previous research [9,11,37]. Research suggests that individuals with a greater liking for fat-containing (discretionary) food and its associated sensations may find nutrient-rich food less palatable and thus consume greater amounts discretionary foods [11]. Future research should extend the range of correlates examined as per an established food choice model [5].

Our findings revealed that young adults who were concordant in discretionary food liking and consumption had higher BMI scores compared with those who were discordant in their liking and intake. We did not identify an association with BMI between those who were concordant and discordant for liking of encouraged core foods. This is in contrast with previous research, where participants who were discordant in fat liking and intake had significantly higher reported BMI scores compared with participants who were concordant [8]. In contrast, BMI did not differ significantly between those who were concordant versus discordant for liking and consuming high-fiber foods [8]. Although this study did not measure dietary restraint specifically, discordance between reported liking and diet quality (reported consumption) could serve as an indirect measure of this construct, as suggested by Duffy et al. [8]. While our findings suggest that measuring agreement between liking and intake of discretionary foods may be useful for assessing differences in BMI, these findings may be a consequence of the participants being a healthy cohort of young adults. Further research is needed to determine whether young adults attempting to limit their consumption of certain foods may be more at risk of emotional over-eating of discretionary foods [37,38,42].

The present study has implications for the design of dietary interventions for use in young adults. Firstly, our results suggest that food liking measures may offer an appropriate methodology for understanding influences on young adults’ food choices. Given that young adults often report a busy lifestyle and a lack of time to prioritize health behaviours [43,44,45], methodologies for capturing food choices that have a lower participant burden than traditional dietary assessment methodologies are important [46,47]. Secondly, research suggests that young adults are not motivated to improve their future health and instead benefit from health messaging that is tailored to their motivators, including weight and physical appearance [48,49]. Thus, the present study provides further insights into potential targets for the tailoring of dietary interventions, including aspects of food literacy, such as food shopping behaviours. Lastly, our findings highlight the need for future research to explore the use of food liking measures to predict prospective diet–disease relationships and whether the use of food liking measures aids in the understanding of complex eating behaviours, such as disordered eating and emotional eating, that are increasingly prevalent in young adult populations [50,51].

A number of limitations should be acknowledged. The participants were relatively homogenous with regards to food liking, BMI, nationality and age. Participants were also female-skewed and studying a food and nutrition subject, and thus were more likely to have a healthier diet and lifestyle [39]. Moreover, the potential for social desirability biases from students being enrolled in a nutrition course should be taken into consideration when interpreting results. Future research in a larger and more representative sample of Australian adults is warranted. Nonetheless, the large sample size in this study provides a valuable contribution to the food liking literature in young adults. Due to its cross-sectional nature, no causal relationships between food liking and correlates were able to be inferred. Moreover, although analyses were adjusted for other key correlates, results may be subject to residual confounding. The potential of self-reporting biases from the questionnaires used in the present study cannot be discounted. Although height and weight were self-reported, evidence suggests that differences in self-reported vs. measured height and weight are minimal in young adults [52]. While previous research has validated the FFQ [9,19], measurement error due to recall and social desirability bias cannot be discounted. Nevertheless, assessing dietary behaviours using a Food Liking Questionnaire as well as a FFQ has been shown to minimize the impact of dietary measurement error [44,46]. The Food Liking Questionnaire did not specify ingredients of food preparation techniques; therefore, it is reasonable to assume that foods such as a “toasted sandwich” or “pizza” might be classified in more than one category depending on which ingredients are used and how it is prepared. Certain items in the Food Liking Questionnaire may also have been interpreted differently by differing participants. Nonetheless, the food liking questionnaire has been validated by Wanich et al. [19] and was found to have a test–retest reliability of moderate to excellent. Future research should examine how best to refine and or adjust this food liking measure for best use as an alternative measure of intake and health outcomes.

The present study aimed to identify correlates of food liking in young adults. Young adults with a higher liking for encouraged foods were older, female, had healthier behaviors (including food shopping and consuming less packaged foods), and had better diet quality. In contrast, a higher liking for discouraged foods and beverages was associated with less healthy behaviours, such as smoking, higher BMI and lower diet quality. These results suggest that food liking measures may offer an appropriate methodology for understanding influences on young adults’ food choices. Furthermore, the differences in food liking scores by participant characteristics further support the tailoring of dietary interventions in young adults. Future research should extend these findings to a wider range of correlates within the wider food choice model, and should investigate whether the use of food liking measures will help understand prospective diet–disease relationships and complex eating behaviours, such as disordered eating and emotional eating, that are increasingly prevalent in young adult populations.

## Figures and Tables

**Table 1:** Characteristics of young Australian adults (*n* = 1728).

	Mean (SD) or Percentage
Demographics	
Age, years	21.8 (6.0)
Female	76.1
Australian nationality	82.6
Living away from home	28.1
Maternal education	
Low	39.6
Middle	22.5
High	38.0
Paternal education	
Low	33.5
Middle	30.2
High	36.4
Health behaviours	
Smoker	5.1
BMI, kg/m^2^	22.9 (3.5)
Do food shopping	46.2
Cook meals	20.8
Main meal convenience/pre-packaged foods	
Never	42.3
Less than one/week	30.9
More than once/week	26.9
Diet	
Self-rated diet	
Unhealthy	13.3
Healthy	67.4
Very healthy	19.3
Diet quality	94.2 (12.3)

Diet quality estimated using the Dietary Guideline Index; education: low (no formal qualifications, Year 10 or equivalent and Year 12 or equivalent), middle (trade/apprenticeship, certificate/diploma), or high (university degree, higher university degree).

**Table 2:** Cronbach’s Alphas and mean food liking scores for encouraged core and discretionary foods in young Australian adults (*n* = 1728).

Food Liking Group	Cronbach’s Alpha	Mean (SD) Food Liking Score
Total encouraged core foods	0.86	6.73 (0.81)
Grains	0.69	6.64 (1.10)
Vegetables	0.80	6.73 (1.14)
Fruit	0.71	7.60 (0.93)
Plant-based proteins	0.74	6.18 (1.61)
Animal-based meat and alternatives	0.91	6.31 (1.92)
Dairy	0.79	6.73 (1.88)
Total discretionary foods	0.92	6.40 (1.34)
High-salt discretionary foods	0.88	6.38 (1.43)
High added sugars discretionary foods	0.84	6.44 (1.44)
Alcoholic beverages	0.73	4.29 (2.15)
Fats and oil	0.49	5.75 (1.45)

**Table 3:** Multiple linear regression analysis for the association of liking encouraged core foods with demographics, health behaviours, diet quality and anthropometrics in young Australian adults (*n* = 1728).

Characteristic	Total Encouraged Core Foods			Grains			Vegetables			Fruit		
	Coeff	95% CI	*p*-Value	Coeff	95% CI	*p*-Value	Coeff	95% CI	*p*-Value	Coeff	95% CI	*p*-Value
Demographics												
Age	0.02	0.01, 0.03	<0.001	0.02	0.01, 0.03	<0.001	0.02	0.01, 0.03	<0.001	−0.01	−0.01, 0.01	0.39
Female	0.03	−0.06, 0.12	0.48	0.43	0.31, 0.55	<0.001	0.59	0.47, 0.71	<0.001	0.19	0.09, 0.30	<0.001
Australian nationality	−0.01	−0.11, 0.09	0.80	0.06	−0.07, 0.19	0.36	−0.06	−0.02, 0.06	0.30	−0.08	−0.21, 0.01	0.09
Living away from home	0.15	0.05, 0.25	0.003	0.14	0.01, 0.28	0.040	0.09	−0.04, 0.22	0.17	0.13	0.02, 0.25	0.020
Maternal education												
Middle	−0.01	−0.11, 0.09	0.90	0.01	−0.12, 0.14	0.88	0.09	−0.03, 0.23	0.14	−0.04	−0.16, 0.08	0.52
High	−0.01	−0.09, 0.09	0.95	0.14	0.01, 0.26	0.030	0.11	−0.01, 0.23	0.07	0.07	−0.04, 0.18	0.21
Paternal education												
Middle	−0.06	−0.16, 0.03	0.20	0.01	−0.11, 0.14	0.81	−0.08	−0.20, 0.05	0.24	−0.08	−0.19, 0.03	0.14
High	−0.08	−.018, 0.01	0.09	−0.11	−0.24, 0.02	0.19	−0.14	−0.26, -0.01	0.030	−0.10	−0.22, 0.01	0.07
Health behaviours												
Smoker	0.01	−0.17, 0.17	0.98	−0.01	−0.21, 0.23	0.93	0.15	−0.07, 0.37	0.17	−0.03	−0.22, 0.16	0.77
BMI	0.01	−0.01, 0.01	0.91	−0.01	−0.03, 0.01	0.05	−0.01	−0.03, 0.01	0.040	0.01	−0.01, 0.02	0.60
Food shopping	0.12	0.02, -0.23	0.018	0.17	0.03, 0.30	0.013	−0.06	−0.20, 0.06	0.32	0.15	0.03, 0.26	0.014
Cook meals	−0.12	−0.23, −0.02	0.018	−0.10	−0.24, 0.03	0.15	−0.29	−0.43, -0.15	<0.001	−0.07	−0.19, 0.05	0.27
Main meal packaged food												
Less than one/week	−0.11	−0.21, −0.03	0.011	−0.06	−0.17, 0.06	0.32	−0.15	−0.27, −0.04	0.010	−0.11	−0.21, −0.01	0.044
Once/week or more	−0.12	−0.22, −0.03	0.012	−0.11	−0.24, 0.01	0.08	−0.23	−0.36, −0.11	<0.001	−0.08	−0.19, 0.03	0.17
Diet												
Self-rated diet												
Healthy	0.24	0.13, 0.36	<0.001	0.37	0.22, 0.53	<0.001	0.30	0.15, 0.45	<0.001	0.21	0.07, 0.34	0.003
Very healthy	0.34	0.20, 0.49	<0.001	0.44	0.25, 0.63	<0.001	0.60	0.42, 0.78	<0.001	0.42	0.25, 0.59	<0.001
Diet quality	0.01	0.01, 0.01	0.001	0.02	0.01, 0.02	<0.001	0.01	0.01, 0.01	<0.001	0.01	0.01, 0.01	<0.001

CI, Confidence Interval; Coeff, Coefficient; BMI, Body Mass Index. Diet quality estimated using the Dietary Guideline Index Multivariate linear regression models were run for each food liking group separately (dependent variable) and included all demographics, lifestyle behaviours anthropometrics and diet quality (independent variables) in the model. Coefficients represent the change in food liking relative to a one-unit change in a correlate, holding all other correlates constant.

**Table 4:** Multiple linear regression analysis for the association of liking core foods and total discretionary foods with demographics, health behaviours, diet quality and anthropometrics in young Australian adults (*n* = 1911).

Characteristic	Legumes and Plant-Based Meal Alternatives			Meat and Animal-Based Alternatives			Dairy			Total Discretionary Foods		
	Coeff	95% CI	*p*-Value	Coeff	95% CI	*p*-Value	Coeff	95% CI	*p*-Value	Coeff	95% CI	*p*-Value
Demographics												
Age	0.03	0.02, 0.04	<0.001	0.03	0.02, 0.05	<0.001	0.03	0.01, 0.05	<0.001	−0.01	−0.02, 0.01	0.26
Female	0.43	0.26, 0.61	<0.001	−0.99	−1.21, −0.78	<0.001	−0.58	−0.79, −0.37	<0.001	−0.23	−0.37, −0.09	0.001
Australian nationality	−0.09	−0.28, 0.09	0.31	0.11	−0.12, 0.34	0.33	−0.06	−0.29, 0.16	0.57	0.04	−0.11, 0.19	0.62
Living away from home	0.11	−0.08, 0.31	0.27	0.22	−0.02, 0.46	0.08	0.30	0.06, 0.54	0.012	0.09	−0.06, 0.25	0.26
Maternal education												
Middle	0.19	−0.01, 0.39	0.05	−0.14	−0.39, 0.09	0.24	−0.18	−0.42, 0.06	0.14	−0.11	−0.26, 0.05	0.19
High	0.15	−0.02, 0.33	0.09	−0.31	−0.53 −0.08	0.006	−0.21	−0.43, 0.01	0.06	−0.06	−0.20, 0.08	0.40
Paternal education												
Middle	−0.18	−0.37, 0.01	0.06	−0.01	−0.24, 0.22	0.93	−0.15	−0.38, 0.08	0.20	−0.02	−0.17, 0.13	0.83
High	−0.03	−0.22, 0.15	0.72	0.01	−0.22, 0.25	0.90	−0.15	−0.38, 0.08	0.20	−0.07	−0.22, 0.08	0.38
Health behaviours												
Smoker	−0.19	−0.52, 0.13	0.23	−0.11	−0.51, 0.29	0.58	0.13	−0.27, 0.52	0.53	0.13	−0.13, 0.39	0.32
BMI	−0.04	−0.06, −0.02	<0.001	0.03	0.01, 0.06	0.014	0.03	0.01, 0.06	0.021	0.04	0.02, 0.06	<0.001
Food shopping	−0.16	−0.36, 0.03	0.10	0.29	0.05, 0.53	0.019	0.47	0.23, 0.71	<0.001	0.39	0.23, 0.55	<0.001
Cook meals	−0.31	−0.51, −0.11	0.002	−0.01	−0.25, 0.24	0.97	0.12	−0.13, 0.36	0.34	0.11	−0.05, 0.28	0.16
Main meal packaged food												
Less than one/week	−0.19	−0.37, −0.02	0.030	−0.14	−0.35, 0.07	0.20	0.09	−0.12, 0.30	0.40	0.32	0.18, 0.46	<0.001
Once/week or more	−0.17	−0.36, 0.01	0.06	−0.12	−0.35, 0.11	0.32	0.19	−0.04, 0.42	0.11	0.51	0.36, 0.66	<0.001
Diet												
Self-rated diet												
Healthy	0.34	0.12, 0.57	0.002	0.11	−0.17, 0.38	0.44	0.09	−0.18, 0.35	0.53	−0.04	−0.22, 0.14	0.66
Very healthy	0.81	0.53, 1.09	<0.001	−0.02	−0.36, 0.33	0.92	−0.44	−0.78, -0.10	0.011	−0.69	−0.92, −0.47	<0.001
Diet quality	0.02	0.01, 0.02	<0.001	−0.02	−0.02, -0.01	<0.001	0.01	−0.01, 0.01	0.76	−0.02	−0.02, −0.01	<0.001

CI, Confidence Interval; Coeff, Coefficient; BMI, Body Mass Index. Diet quality estimated using the Dietary Guideline Index Multivariate linear regression models were run for each food liking group separately (dependent variable) and included all demographics, lifestyle behaviours, anthropometrics and diet quality (independent variables) in the model. Coefficients represent the change in food liking relative to a one-unit change in a correlate, holding all other correlates constant.

**Table 5:** Multiple linear regression analysis for the association of liking discretionary foods with demographics, health behaviours, diet quality and anthropometrics in young Australian adults (*n* = 1911).

Characteristic	High-Salt Discretionary Foods			High Added Sugar Discretionary Foods			Alcoholic Beverages			Fats and Oil		
	Coeff	95% CI	*p*-Value	Coeff	95% CI	*p*-Value	Coeff	95% CI	*p*-Value	Coeff	95% CI	*p*-Value
Demographics												
Age	−0.01	−0.02, 0.01	0.47	−0.01	−0.02, 0.01	0.14	0.08	0.07, 0.10	<0.001	0.03	0.01, 0.04	<0.001
Female	−0.28	−0.43, −0.13	<0.001	−0.16	−0.30, -0.01	0.044	−0.43	−0.67, −0.19	<0.001	−0.22	−0.38, −0.05	0.01
Australian nationality	0.09	−0.06, 0.25	0.26	−0.03	−0.20, 0.13	0.67	0.08	−0.18, 0.34	0.54	−0.18	−0.36, −0.01	0.045
Living away from home	0.07	−0.09, 0.24	0.42	0.12	−0.05, 0.29	0.17	0.43	0.17, 0.70	0.001	0.07	−0.11, 0.26	0.44
Maternal education												
Middle	−0.05	−0.22, 0.12	0.56	−0.18	−0.36, −0.01	0.036	0.04	−0.23, 0.31	0.79	−0.07	−0.26, 0.11	0.42
High	−0.06	−0.22, 0.09	0.42	−0.06	−0.21, 0.09	0.47	0.17	−0.07, 0.42	0.16	−0.05	−0.22, 0.11	0.54
Paternal education												
Middle	−0.05	−0.21, 0.11	0.51	0.03	−0.13, 0.19	0.68	0.28	0.03, 0.54	0.03	−0.08	−0.26, 0.09	0.35
High	−0.10	−0.26, 0.05	0.20	−0.01	−0.18, 0.15	0.86	0.32	0.06, 0.58	0.01	−0.08	−0.26, 0.09	0.37
Health behaviours												
Smoker	0.18	−0.09, 0.46	0.20	0.06	−0.22, 0.35	0.66	0.52	0.08, 0.96	0.021	0.09	−0.21, 0.40	0.54
BMI	0.04	0.02, 0.06	<0.001	0.04	0.02, 0.06	<0.001	−0.01	−0.04, 0.02	0.50	0.02	0.01, 0.04	0.025
Food shopping	0.40	0.23, 0.57	<0.001	0.37	0.19, 0.54	<0.001	−0.24	−0.51, 0.03	0.081	0.30	0.12, 0.49	0.001
Cook meals	0.08	−0.09, 0.25	0.37	0.16	−0.01, 0.34	0.070	−0.13	−0.41, 0.14	0.35	−0.20	−0.39, −0.01	0.037
Main meal packaged food												
Less than one/week	0.31	0.16, 0.46	<0.001	0.34	0.19, 0.49	<0.001	0.03	−0.20, 0.27	0.79	0.14	−0.02, 0.30	0.09
Once/week or more	0.49	0.33, 0.65	<0.001	0.54	0.37, 0.70	<0.001	0.11	−0.14, 0.36	0.40	0.31	0.13, 0.48	0.001
Diet												
Self-rated diet												
Healthy	−0.01	−0.20, 0.18	0.91	−0.08	−0.27, 0.12	0.44	0.12	−0.19, 0.42	0.46	0.12	−0.09, 0.33	0.25
Very healthy	−0.72	−0.96, −0.48	<0.001	−0.66	−0.91, −0.42	<0.001	−0.03	−0.41, 0.35	0.87	−0.27	−0.54, −0.01	0.044
Diet quality	−0.02	−0.02, −0.01	<0.001	−0.01	−0.02, −0.01	<0.001	−0.01	−0.01, 0.004	0.34	−0.01	−0.02, −0.01	<0.001

CI, Confidence Interval; Coeff, Coefficient; BMI, Body Mass Index. Diet quality estimated using the Dietary Guideline Index. Multivariate linear regression models were run for each food liking group separately (dependent variable) and included all demographics, lifestyle behaviours, anthropometrics and diet quality (independent variables) in the model. Coefficients represent the change in food liking relative to a one-unit change in a correlate, holding all other correlates constant.

## Supplementary Materials

The following are available online at https://www.mdpi.com/2072-6643/12/10/3078/s1 Table S1: Food liking groups created from the Food Liking Questionnaire; Table S2: Correlations between food liking groups
