# Supplementary material for: Demographic, Behavioural and Anthropometric Correlates of Food Liking: A Cross-sectional Analysis of Young Adults
Source: Nutrients. 2020 Oct 9;12(10):3078. doi: 10.3390/nu12103078 (PMC7601355; doi:10.3390/nu12103078)
Supplement: Supplementary file 1 [file nutrients-12-03078-s001.pdf]

**Supplemental Table 1**: Food liking groups created from the Food Liking Questionnaire

| Food groups (124 items) | Food Items |
|---|---|
| Grains (7 items) | Plain porridge, wholegrain bread, spaghetti, rice, grains, muesli, quinoa |
| Vegetables (10 items) | Tomato, greens, broccoli, carrot, cabbage, mushrooms, potato (not deep-fried chips), vegetable soup, capsicum/ pepper (not hot chilli), brussels sprout |
| Fruits (7 items) | Apple, pineapple, melon, berries, banana, orange, grapes |
| Legumes and plant-based alternatives (4 items) | Beans and beans products (not including beverages), tofu, nuts, lentils |
| Meat and animal-based alternatives (9 items) | Beef steak, lamb, pork products, chicken, duck, white fish, pink fish, eggs, turkey e.g. breast, slices |
| Dairy (3 items) | Milk, yoghurt, cheese |
| High-salt discretionary foods (11 items) | Cornflakes, white bread, potato chips (crisps), corn chips, savoury biscuits, hamburgers, hot chips, Asian takeaway, pizza, toasted sandwich, KFC/ Red Rooster/ rotisserie chicken |
| High-added sugars discretionary foods (8 items) | Ice cream, sweet biscuits, chocolate, lollies, cake, cola soft drinks, citrus soft drinks, fruit juice |
| Alcoholic beverages (3 items) | Red wine, white wine, beer e.g. lager/ bitter |
| Fats and oil (3 items) | Butter, margarine, olive oil |
| Total encouraged core foods - includes grains, vegetables, fruits, plant-based proteins, animal-based proteins and dairy) (40 items) | Plain porridge, wholegrain bread, spaghetti, rice, grains, muesli, quinoa, beef steak, lamb, pork products, chicken, duck, white fish, pink fish, eggs, turkey e.g. breast, slices, beans and beans products (not include beverages), tofu, nuts, lentils, tomato, greens, broccoli, carrot, cabbage, mushrooms, potato (not deep fried chips), vegetable soup, capsicum/ pepper (not hot chilli), brussels sprout, apple, pineapple, melon, berries, banana, orange, grapes, milk, yoghurt, cheese |
| Total discretionary foods – includes high salt and high added sugar discretionary foods (19 items) | Cornflakes, white bread, potato chips (crisps), corn chips, savoury biscuits, hamburgers, hot chips, Asian takeaway, pizza, toasted sandwich, KFC/ Red Rooster/ rotisserie chicken, ice cream, sweet biscuits, chocolate, lollies, cake, cola soft drinks, citrus soft drinks, fruit juice |

**Supplemental Table 2**: Correlations between food liking groups

| | Total core encouraged foods | Grains | Vegetables | Fruit | Legumes/ plant-based alternatives | Meat/ animal-based alternatives | Dairy | Total discretionary | High-salt discretionary | High-sugar discretionary | Alcoholic beverages | Fats/ Oil |
|---|---|---|---|---|---|---|---|---|---|---|---|---|
| Total core encouraged foods | 1.00 | | | | | | | | | | | |
| Grains | 0.61 | 1.00 | | | | | | | | | | |
| Vegetables | 0.65 | 0.46 | 1.00 | | | | | | | | | |
| Fruit | 0.55 | 0.40 | 0.43 | 1.00 | | | | | | | | |
| Legumes/ plant-based alternatives | 0.50 | 0.47 | 0.58 | 0.29 | 1.00 | | | | | | | |
| Meat/ animal-based alternatives | 0.61 | 0.03 | -0.02 | 0.04 | -0.12 | 1.00 | | | | | | |
| Dairy | 0.49 | 0.12 | -0.03 | 0.12 | -0.07 | 0.53 | 1.00 | | | | | |
| Total discretionary | 0.23 | 0.09 | -0.14 | 0.11 | -0.21 | 0.36 | 0.47 | 1.00 | | | | |
| High-salt discretionary | 0.22 | 0.11 | -0.13 | 0.08 | -0.20 | 0.35 | 0.43 | 0.96 | 1.00 | | | |
| High-sugar discretionary | 0.21 | 0.07 | -0.14 | 0.13 | -0.18 | 0.32 | 0.46 | 0.91 | 0.76 | 1.00 | | |
| Alcoholic beverages | 0.29 | 0.17 | 0.19 | 0.09 | 0.17 | 0.19 | 0.17 | 0.14 | 0.15 | 0.11 | 1.00 | |
| Fats and Oil | 0.36 | 0.12 | 0.05 | 0.07 | -0.03 | 0.41 | 0.49 | 0.51 | 0.49 | 0.47 | 0.22 | 1.00 |

Pearson correlation coefficients were used to assess correlations
